# MEDICARE COVERAGE CHANGES: THE ROLE OF INDIVIDUAL CHARACTERISTICS AND INTERNET USE

**DOI:** 10.1093/geroni/igz038.1100

**Published:** 2019-11-08

**Authors:** Brian E McGarry, David C Grabowski

**Affiliations:** Harvard Medical School, Boston, Massachusetts, United States

## Abstract

An important challenge to the success of consumer choice within the Medicare program is older adults’ proclivity to assess their coverage on an annual basis and make changes when appropriate. Every year, relatively few beneficiaries alter their coverage (e.g., by switching Part D plans, switching between MA and traditional Medicare coverage) despite annual coverage changes and new market entrants. Little is known about the factors that encourage re-evaluation of Medicare coverage choices or the role of technology in facilitating changes. The latter knowledge gap is particularly relevant as the internet is an increasingly important delivery mechanism for Medicare information and consumer support tools. This study uses a nationally-representative sample of Medicare beneficiaries to describe Medicare coverage changes, the individual factors that predict such changes, and the relationship between Medicare-related internet use and plan switching. On average, 12% of Medicare beneficiaries made changes to their coverage in a given year, with 25% of beneficiaries making a change at any point during the study period. Between 2011 and 2015, the self-reported rate of using the internet to handle Medicare/insurance matters increased from 5% to 11%. In adjusted models that included individual-level fixed effects and other time-varying characteristics (e.g., health status, prescription drug needs), Medicare-related internet use was associated with a 65% increase in the probability of making a coverage change. Although using the internet to handle insurance matters remains relatively rare among older adults, it may an important mechanism for obtaining information that encourages plan changes, facilitating such switches, or both.

